# Comment on “Individual and Neighborhood Socioeconomic Status and the Association between Air Pollution and Cardiovascular Disease”

**DOI:** 10.1289/EHP852

**Published:** 2017-01-01

**Authors:** Tara Ahmed, Heval Mohamed Kelli, Pratik Sandesara

**Affiliations:** 1 Schulich School of Medicine & Dentistry, Western University, London, Ontario, Canada; 2 Emory Clinical Cardiovascular Research Institute, Emory University, Atlanta, Georgia, USA

We read with great interest Chi et al.’s article examining the role of socioeconomic status (SES) in the air pollution–cardiovascular disease (CVD) relationship. We appreciate their consideration of multiple levels of SES in this relationship. The authors have paved the path to consider similar studies on other populations. However, we note that, given the composition of the study population, the findings of this report can be generalized only to a subset of the population who are female, white, postmenopausal, over 50 years old, and free of CVD at baseline. In order for the findings to apply to the general population, potential differences related to factors such as sex and race would need to be considered.

As CVD research has grown to include more female participants, significant sex differences have recently been found. Atypical presentation of CVD is more prevalent in women than in men (Canto et al. 2012; Ski et al. 2014). Moreover, the sex disparity in evidence-based treatment can lead to delayed intervention and adverse cardiovascular outcomes (Ski et al. 2014). In addition, the risk for cardiovascular complications in diabetics is higher in women than in men (Huxley et al. 2006; Maas and Appelman 2010). Since there are substantial sex differences in CVD, there may also be sex differences in the role of SES in the association between air pollution and CVD.

While the article provides relatively recent findings, its applicability to current populations is limited due to the data collection timeline. Participants were initially enrolled between 1993 and 1998. This enrollment period occurred before a crucial turning point in health care, specifically the 1999 release of the first woman-specific clinical recommendations by the American Heart Association (Lewis et al. 2009; Mosca et al. 2011; Ski et al. 2014). Since the release of the recommendations, there have been major improvements and changes in risk factor awareness, prevention, and treatment of CVD in women (Lewis et al. 2009; Mosca et al. 2011; Ski et al. 2014). Furthermore, in 2004 the American Heart Association published the first evidence-based guidelines for female CVD prevention, and the rate of death from CVD among women decreased by nearly half between 1997 and 2009 (Mosca et al. 2011). Therefore, if the same study were conducted today, we speculate that the findings might be different based on these more recent improvements in women’s CVD awareness, prevention, and treatment.

In addition to sex differences, notable differences in CVD have been associated with racial and ethnic differences. CVD, related risk factors, and mortality have occurred at a higher prevalence in black individuals than in white individuals (Feinstein et al. 2012; Ski et al. 2014). For example, the prevalence of CVD in black women is 47%, compared with 34% in white women (Mosca et al. 2011; Roger et al. 2011). In 2007 the rate of hypertension as a cause of death in black women was nearly double the rate in white women (Mosca et al. 2011; Roger et al. 2011). These differences emphasize the need to consider potential racial disparities, as well as sex disparities, when examining the role of SES in the air pollution–CVD relationship.

Finally, Chi et al. excluded 18,576 participants for having CVD at baseline. While their findings have implications for preventing CVD outcomes in individuals who initially lack CVD, it would also be informative to conduct a similar study in those who do have CVD at baseline. Such a study could yield insight into individuals’ susceptibility to progression of CVD, related hospitalizations or mortality, comorbidities, and overall health.
